# Cancer Care in 2016: The ASCO State of Cancer Care Report and the Role of Advanced Practitioners

**DOI:** 10.6004/jadpro.2016.7.5.1

**Published:** 2016-07-01

**Authors:** Pamela Hallquist Viale

The American Society of Clinical Oncology published their annual State of Cancer Care in America: 2016 report in March of this year (ASCO, 2016). As expected, the report detailed the many improvements in care as well as the challenges and problems that remain in cancer care in the United States today. I’d like to give you a summary picture of the report and where we stand regarding cancer care in America for 2016.

We continue to see improvements in some survival rates and new treatment breakthroughs. However, although decreased mortality has been achieved in some cancer types (such as breast, colorectal, and cervical cancers), other outcomes remain steady despite new therapies. Bladder cancer, brain cancer, and melanoma continue to have similar mortality rates and the survival rates for pancreatic and liver cancer have actually decreased. Differences in mortality continue to be affected by race and ethnicity (ASCO, 2016).

Approvals of new therapies have added 15 new agents and biologic therapies to our armamentarium, with additional expanded uses for previously approved therapies. It’s exciting to see that the use of immunotherapy has improved outcomes for many patients who previously had no effective therapies; challenges remain with cost of therapy and reimbursement policies (ASCO, 2016). Last year also saw the introduction of our first biosimilar agent, opening the door to further approvals in the future.

Cancer care continues to increase in its complexity and implementation. This year’s report focused on cancer screening, the implementation of precision medicine treatments (as highlighted by President Obama as a needed strategy to improve care), and the aging of our US population. Changes in screening for specific cancers have been controversial and complex as providers aim to reduce over and under-screening of cancers, while strategizing to make the best screening decisions possible. The use of precision medicine assists in further refining optimal treatment by use of appropriate genetic testing, and our aging population creates a care environment in which larger numbers of patients with cancer will continue to have comorbid conditions leading to increased care complexity (ASCO, 2016).

The report discusses the very real problem of increased drug prices and their effects on patients, describing the issues with patients’ rising deductibles and cost shifting (ASCO, 2016). Although the Affordable Care Act (ACA) has provided health insurance to a large number of Americans, problems still exist with coverage for the elderly and for those patients who can’t afford the deductible or out-of-pocket costs associated with cancer care.

## THE EVOLVING WORKFORCE

Workforce trends indicate that oncology practices are experiencing significant changes in the workplace. Although the workforce remains stable, the demand for oncology services continues to grow and oncology specialists continue to age. The cancer care team model is increasingly the choice for care, with providers from different specialties working together to provide an integrated approach to cancer care. You might be wondering where advanced practice providers (APPs) fit within this cancer team model.

The ASCO State of Cancer Care report recognizes that advanced practice providers, including nurse practitioners (NPs) and physician assistants (PAs) are important in cancer care today in the US. The many roles an APP plays are acknowledged in the report, including the ordering of chemotherapy treatments and the provision of pain and symptom management. The report outlines the role of APPs in the provision of routine primary care services for patients with active disease as well as cancer survivors, and notes that almost three-quarters of ASCO Census practices reported an advanced practice provider(s) in their practices (n = 3,913 NPs and 1506 PAs) with an average of 0.44 APP per oncologist (ASCO, 2016). This information helps to validate the growing role of the advanced practice provider in the care of patients with cancer today. Importantly, ASCO recognizes that APPs and their contributions to care are significant. ASCO also notes that further information and data is needed to refine and improve their knowledge base on the number of APPs working in cancer care today as well as increase understanding of the expanding role and duties of the APP. To that end, ASCO is partnering with APSHO and other professional societies to obtain that information—the results will be published in 2017.

The recognition and adaption of team-based care is critical to cancer care services; collaborative care coupled with improvements in technology is essential in increasing quality and efficacy of cancer therapies and services. These improvements in care can be achieved with interprofessional education and strategies to better coordination of care in practices and health care systems (ASCO, 2016).

I encourage all of you to read the entire paper published in the *Journal of Oncology Practice*. As advanced practitioners working in oncology today, we have a recognized and integral role in the care of patients with cancer. It is our responsibility to remain current and active in the improvements of cancer care. As members of APSHO, we have a seat at the table and are participating in the very real decision-making that accompanies changes in health care.

## JADPRO LIVE AT APSHO 2016

Plans for JADPRO Live at APSHO 2016 are underway. I hope to see you in Washington, DC this November for our fourth annual conference and APSHO meeting. If you’ve joined us before, you can expect more of our focused curricula and collaborative educational approach. If you haven’t been to a JADPRO Live at APSHO conference, and plan to attend this year, you are in for a truly practice-changing experience. Our interdisciplinary faculty and agenda aim to provide you with the content you’ll find useful in practice. Visit our website for more information as well as an agenda—I promise you an exciting and relevant meeting!
